# A SAW Wireless Passive Sensing System for Rotating Metal Parts

**DOI:** 10.3390/s24206703

**Published:** 2024-10-18

**Authors:** Yue Zhou, Jing Ding, Bingji Wang, Feng Gao, Shurong Dong, Hao Jin

**Affiliations:** 1 College of Information Science & Electronic Engineering, Zhejiang University, Hangzhou 310027, China; 3180101383@zju.edu.cn (Y.Z.); dongshurong@zju.edu.cn (S.D.); hjin@zju.edu.cn (H.J.); 2 Shanghai Marine Diesel Engine Research Institute, No.3111 Huaning Road, Minhang District, Shanghai 201108, China

**Keywords:** passive wireless sensing system, rotating metal objects, design of loop antenna integrated with a rotor, SAW sensor tag, SAW antenna

## Abstract

Passive wireless surface acoustic wave (SAW) sensors are very useful for on-site monitoring of the working status of machines in complex environments, such as high-temperature rotating objects. For rotating parts, it is difficult to realize real-time and continuous monitoring because of the unstable sensing signal caused by the continuous change of the relative position of the rotating part to the sensor and shielding of the signal. In our SAW sensing system, we propose a loop antenna integrated with the rotating part to obtain a stable sensing signal owing to its omnidirectional radiation pattern. Methodologies for determining the antenna dimension, system operating frequency, and procedures for designing a SAW sensor tag are discussed in this paper. By fully utilizing the influence of metal rotor on antenna performance, the antenna needs no impedance matching elements while it provides sufficient gain, which equips the antenna with nearly zero temperature drift at a wide temperature-sensing range. Experimental verification results show that this sensing system can greatly improve the stability of the sensing signal significantly and can achieve a temperature sensing accuracy of ~1 °C at different rotational speeds, demonstrated by the feasibility of the loop antenna for monitoring the working status of rotating metal parts.

## 1. Introduction

Some machines, such as turbines and jet engines, operate at extreme temperatures with high rotating speeds; it is therefore important and essential to monitor their working status in real time and on-site to ensure their safe operation [1,2]. Passive wireless surface acoustic wave (SAW) sensing systems have been widely used in applications owing to their low cost, small size, and high operating temperatures [3,4,5,6]. To achieve status monitoring, a SAW sensor tag consisting of a SAW resonator and a small-sized antenna is usually mounted on a target object, such as a rotating part. An RF reader sends pulsed interrogation radio-frequency (RF) signals [7] through another antenna to excite the SAW sensor and receive the echoes from the sensor. The echoed signal achieves the strongest intensity when the interrogation frequency matches the SAW resonant frequency. Variables such as temperature or strain variations may induce a slight shift in the echoed frequency, and can thus be obtained by sweeping the frequency of the interrogation signals to find the resonant frequency of the SAW sensor.

However, it is very challenging to achieve stable and continuous monitoring for rotating metal parts using SAW sensor tags. The fast rotation speed and narrow metal environment result in strong electromagnetic (EM) shielding, multipath interferences, and significant influence on antenna performance [8]. The intensity of the echo signal changes with the relative position of the antenna and the sensor, which rotates with the working part. This variation will interfere with the echo signal as the frequency sweep takes time, which decreases sensing accuracy.

The EM shielding issue can be solved by distributed antenna array technology [9,10,11]. By placing antennas around a rotating object, multiple paths to the sensor tag can be established. As long as one path is not shielded, the signal connection can be maintained. Sophisticated synchronization between the antenna array [12] and rotating part is required in this approach to achieve accurate synthesis of the received echoes from each antenna. Sensors based on a potentiometer, optics, or the Hall effect [13,14,15] are typically used for rotation angle measurement and synchronization, but these sensors are usually high-temperature intolerant [16]. Moreover, installation of the antenna array and additional sensors often has high costs and can also affect the safe operation of the machine. 

Multipath interference affects the signal integrity of the interrogation echoes and reduces the sharpness of the obtained SAW resonance peak, which will reduce the frequency resolution of the sensor. A multi-input multi-output (MIMO) antenna array has been proposed for overcoming this problem [17,18]. An MIMO enables a beam of the interrogation signal to form and establishes a point-to-point direct link to the sensor, which suppresses the generation of multipath interference. However, this requires beam tracking of the sensor tag during the object rotation. This requires complex hardware systems and algorithms. Most MIMO antenna arrays are designed for systems operating over several gigahertz; the antenna dimension could be very large if this technique is adopted in passive wireless SAW sensor systems that commonly operate within hundreds of megahertz. Moreover, an MIMO cannot solve the issue of EM shielding and even aggravates the problem due to its high directivity.

An alternative solution to the above problem is to use a large-diameter RF rotary coupler for near-field wireless interrogation [19]. It has been reported that the passive wireless SAW sensor system with an RF rotary coupler works stably for contactless torque measurements of wind turbines, automotive engines, and other industrial devices [20,21,22]. Average smoothing algorithms are applied for eliminating measurement errors caused by the vibration of the rotating parts. However, this method only works in the near-field, within a range of a few centimeters; as the wireless distance increases, its coupling efficiency decreases dramatically, and the echo signal of the SAW sensor is drawn into noise.

**Figure 1:**
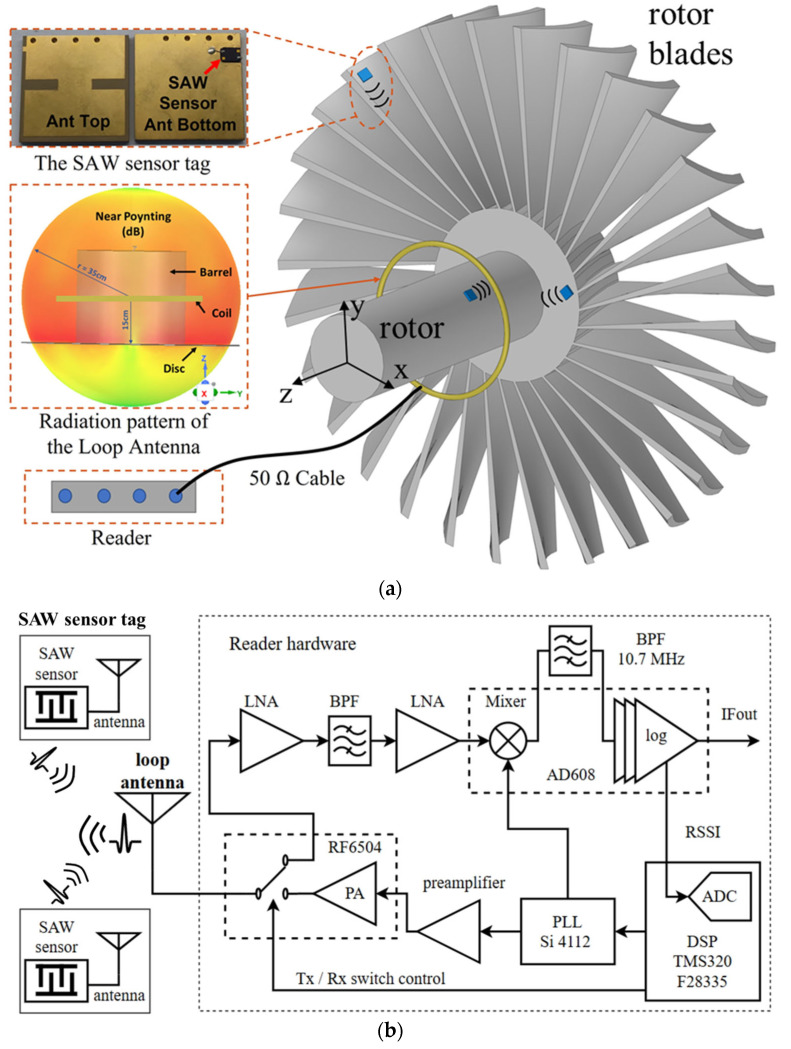
(**a**) System configuration diagram: installation of the loop antenna, SAW sensor tags, and reader; (**b**) Hardware architecture of the reader.

In this paper, we propose a SAW wireless passive sensing system that overcomes the above challenges. The system can be applied to continuous real-time monitoring of the rotor temperature. The diagram in Figure 1a illustrates that the system consists of one or several SAW sensor tags with miniaturized designs mounted on a rotor for temperature sensing, a loop antenna with an omnidirectional radiation pattern around the rotating object, and a reader for sending and receiving RF interrogation signals. 

Figure 1b shows a basic schematic diagram of the reader hardware. Heterodyne architecture was applied to the reader for better noise performance and lower costs. The interrogation signal was synthesized by a phase-locked-loop (PLL) chip and was pre-amplified to drive the power amplifier (PA) in RF6504, a RF front-end chip. The RF switch in this chip controls the timing sequence of transmission and reception. The hardware transmits a burst interrogation signal for 30 μs and then switches to receiving mode. The received echo signal passes through two low-noise amplifiers (LNA) with 20 dB gain each, a noise reduction bandpass filter (BPF), and is down-converted to a 10.7 MHz IF signal with 24 dB conversion gain. The logarithmic limiting amplifier in AD608 provides a received signal strength indicator (RSSI) output with an 80 dB dynamic range. The RSSI output is sampled by an analog-to-digital converter (ADC) on a digital signal processing (DSP) chip. Finally, the resonant frequency of the SAW sensor tag is derived by averaging and fitting these sampled data.

## 2. Theories and System Design Methods

In Section 2, the design theories and methodologies of the loop antenna and SAW sensor tag are researched and discussed. The loop antenna, the turbine rotor, and the SAW sensor tag should be considered as an inseparable system rather than individual components. Design parameters such as the system operating frequency, antenna impedance, the gain (efficiency) and miniaturization factor, the bandwidth and temperature sensing range, the temperature coefficients of antennas, etc., are all interrelated. Hence, these parameters should be collaboratively considered to obtain an optimal solution for the passive wireless sensing system on rotating metal parts.

Section 2.1 mainly discusses the optimization goals of the loop antenna integrated with the turbine rotor. A simple turbine rotor model was established to analyze the characteristics of the loop antenna. The validation of the model was analyzed by simulating the distribution of the eddy current and magnetic field. 

Normalized methodologies and an empirical formula for optimizing the loop antenna impedance in the harsh environment of the turbine rotor were proposed by analyzing the influence of the turbine rotor and comparing that with the loop antenna in free space. Normalized look-up curves were displayed for quickly finding the geometric size of the loop antenna. According to the proposed method, a loop antenna operating in different frequency bands can be designed, and its operating frequency can be continuously adjusted. The antenna can achieve a good voltage standing wave ratio (VSWR) without any additional impedance, matching components by using the proposed methods.

Antenna radiation patterns were simulated to obtain information on enhancing the wireless sensing distance. The rotor blades are utilized as a reflector, and a suitable distance between the blades and the loop antenna is adopted to obtain higher gain. The far-field gain and the near-field Poynting energy flow were displayed in this section to determine the mounting position of the SAW sensor tag with good signal strength. The SAW sensor tag can be mounted in either of these regions.

Section 2.2 mainly discusses the design methodologies of the SAW sensor tag, including the SAW antenna (i.e., antenna of the SAW sensor tag) and the SAW temperature sensor. Different architectures of the SAW antenna are listed and compared, and the reason why planar inverted F antenna (PIFA) is a good candidate for SAW sensor tags operating in metal rotating environments is explained. Miniaturization design methods for PIFA and solutions for suppressing the distortion of its radiation pattern are discussed that ensure the wireless sensing distance is not reduced due to mismatched polarization direction. Design theories and considerations for the optimal performance of the SAW resonator and its fabrication process flow are also introduced in this Section.

The antenna bandwidth and its temperature drift are significant design constraints for both the loop antenna and SAW antenna because they affect the maximum temperature sensing range. The loop antenna was simulated and analyzed at nearly zero temperature drift in Section 2.1. The temperature drift of PIFA was more significant due to the sacrifice of bandwidth in the process of miniaturization. It was suppressed by adopting substrate material with a zero temperature coefficient, introduced in Section 2.2, and was tested, as shown in Section 3. The minimum bandwidth of the antennas must cover the frequency shift of the SAW resonator within a whole temperature sensing range; otherwise, the RF signal will decrease due to the frequency mismatch between the SAW sensor and antennas. Conversely, the sensing range of this system can be estimated by the antenna bandwidth. The estimation method is discussed according to the measurement results in Section 3.

### 2.1. Optimization of the Loop Antenna Performance

In the harsh environment of a turbine rotor, general impedance matching components such as inductors, capacitors, and microstrips are inapplicable for tuning the impedance of the loop antenna due to their unstable performance at high temperatures (usually less than 150 °C) and their large temperature drift. Although microstrips formed on microwave ceramics, such as corundum, have the advantage of high temperature tolerance, they are too long for typical passive wireless sensing system operation at several hundreds of megahertz. Thus, it leads to complex installation and the occupation of too much of an area. A good solution to this problem can be optimizing the loop antenna impedance to 50 Ω without any matching components while maintaining a high gain. The impedance of the loop antenna in free space is firstly analyzed in Section 2.1.1 to obtain some information on this optimization solution.

#### 2.1.1. The Impedance of the Loop Antenna in Free Space

**Figure 2:**
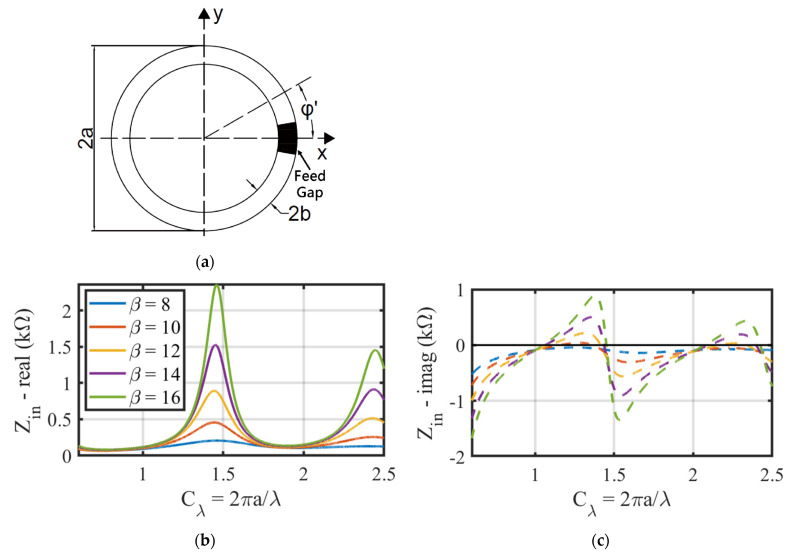
(**a**) The geometric dimensions of a loop antenna; Real (**b**) and imaginary (**c**) part of the loop antenna impedance in free space with different values of *β* and a normalized circumference *C_λ_*.

The impedance of the loop antenna in free space was initially calculated using an analytical method to research its resonant behaviors in this Section. Considering the geometric diagram of the loop antenna placed on the xy plane in Figure 2a, where 2a and 2b are its outer loop diameter and wire diameter, respectively. C and $C_{\lambda }$ are defined to be the loop circumference and normalized circumference (normalized by its operating wavelength, λ), respectively, i.e., $C_{\lambda }=C/\lambda =2\pi a/\lambda$. Then, we use $\beta =2\ln\left(2\pi a/b\right)$ to characterize the ratio of the antenna circumference to the wire diameter.

The azimuth angle $\varphi^{\prime }$ in Figure 2a denotes the angular coordinate on the loop. A feed gap is designed at $\varphi^{\prime }=0$ with an excitation voltage of V. According to [23], when the loop radius is comparable with its operating wavelength, the current in the loop can be approximated by a Fourier series, as shown by Equation (1):

(1) $$I\left(\varphi^{\prime }\right)=\sum_{n=0}^{\infty }I_{n}\cos\left({n\varphi }^{\prime }\right)=\frac{V}{j\pi \eta_{0}}\left[\frac{1}{c_{0}}+2\sum_{n=1}^{\infty }\frac{\cos\left({n\varphi }^{\prime }\right)}{c_{n}}\right]$$

where $\eta_{0}$ is the vacuum wave impedance (120π Ω). The input impedance $Z_{in}$ of the loop antenna is the excitation voltage of V divided by the current at the feed gap, i.e., $\varphi^{\prime }=0$. Thus, according to Equation (1), $Z_{in}$ is expressed as follows:

(2) $$Z_{in}=\frac{V}{I\left(\varphi^{\prime }=0\right)}=j\pi \eta_{0}{\left[\frac{1}{c_{0}}+2\sum_{n=5}^{\infty }\overset{-1}{\underset{n}{c}}\right]}^{-1}$$

It was reported in [23] that the number of terms in Equation (2) for convergence is $max(5,\;3C_{\lambda }).$ When $0<C_{\lambda }<2.5$, only the first four terms in the summation of Equation (2) need to be calculated explicitly. From the $5^{th}$ term, the summation can be approximated by the integral in Equation (3) with 1% accuracy:

(3) $$\sum_{n=5}^{\infty }c_{n}^{-1}=\pi C_{\lambda }\left[\frac{1}{n_{0}}\int_{0}^{\ln\left(\frac{2ae^{-\gamma }}{4.5b}\right)}x^{-1}e^{x}dx+\frac{C_{\lambda }^{2}}{273.375}\ln^{-1}\left(\frac{2ae^{-\gamma }}{4.5b}\right)\right]$$

The curves in Figure 2b,c show the calculated real and imaginary parts of the loop antenna impedance at various β. Two self-resonance points appear at $C_{\lambda }=1.05$ and $C_{\lambda }=2.1$ for β=14, and their real parts of $Z_{in}$ are 141 Ω and 200 Ω, correspondingly. At $C_{\lambda }=1.4$ and $C_{\lambda }=2.4$, two anti-resonance points occur with large real part impedances up to thousands of ohms, which means the antenna cannot absorb the power energy delivered by the reader.

It can be concluded from Figure 2b,c that for all values of β, the self-resonant point of the loop antenna in free space appears close to multiple integers of $C_{\lambda }$, while the anti-resonant point appears in the middle of two adjacent integers of $C_{\lambda }$. The antenna radiation resistance, i.e., the real part of $Z_{in}$, is within the range of 100 Ω to 300 Ω at self-resonant points. While at anti-resonant points, the antenna radiation resistance is approximately ten times larger than that at self-resonant points, so it is easier to optimize the antenna radiation resistance to 50 Ω at self-resonant points of the loop antenna.

Hence, the operating frequency of the loop antenna is determined at its self-resonant frequency rather than its anti-resonant frequency. According to these results, a rough but simple empirical formula for designing a loop antenna at its self-resonant points at any operating frequency can be expressed by Equations (4) and (5) as follows:

(4) $$2\pi a\approx k\lambda ,\left(k=1,2,\ldots \right)$$

(5) $$real\left(Z_{in}\right)\in \left(100,300\right)$$

Four 400 MHz (λ=75 cm) loop antennas in free space with k=1,2,3,4 were designed using Equation (4) and simulated using a 3D electromagnetic simulator (Ansys HFSS) to verify the accuracy of the equations above. The comparison results are given in Table 1, where the maximum estimation error for calculating its self-resonant frequency is less than 7%, and the simulated radiation resistance of all antennas is within the range of calculation as well. The positive frequency error can be easily trimmed to the design value by slightly increasing the antenna circumference.

To optimize the antenna radiation resistance to 50 Ω without any impedance matching components, the impact of the turbine rotor on the antenna performance, including its self-resonant frequency, radiation impedance, and radiation pattern, is discussed in the following sections.

#### 2.1.2. Loop Antenna Integrated with a Rotor 

**Figure 3:**
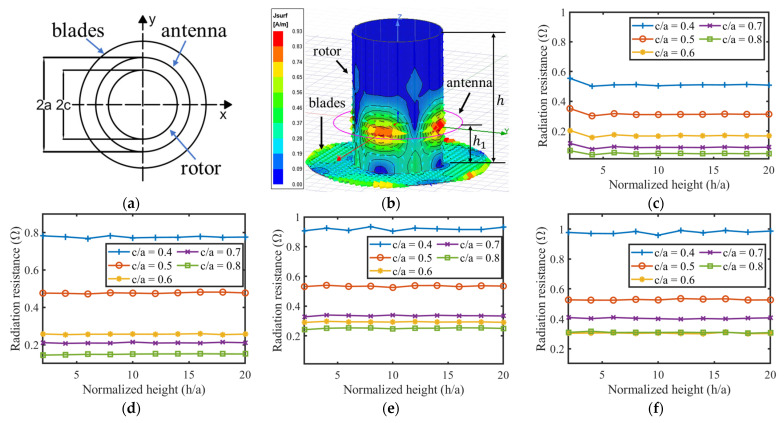

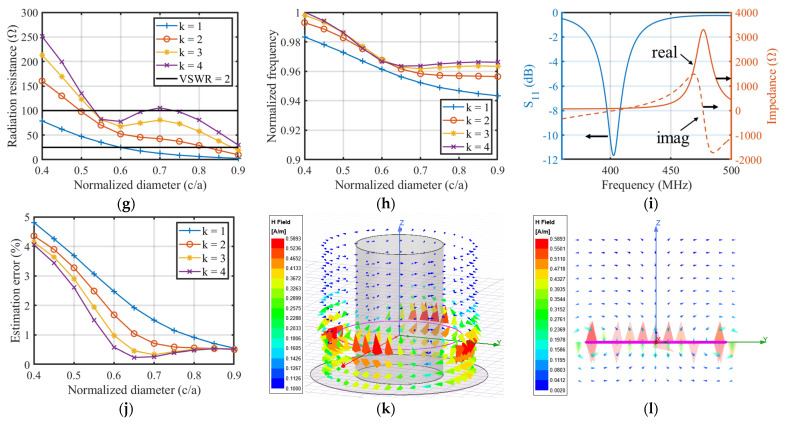
(**a**) The top view projection and (**b**) the three-dimensional view of the simulation model together with the simulated surface current distribution of the model when k=2; (**c**–**f**) The relationship between normalized antenna radiation resistance, rotor height (h/a), and rotor diameter (c/a) of four loop antenna with k=1,2,3,4, respectively; (**g**) Antenna radiation resistance and (**h**) normalized antenna self-resonant frequently at different normalized rotor diameters (c/a); (**i**) Simulated return loss ($S_{11}$ parameter) and impedance spectrum ($Z_{11}$ parameter) of the 400 MHz loop antenna integrated with a rotor; (**j**) Estimation error of Equation (4) for the loop antenna integrated with the rotor model; Magnetic field distribution around the loop antenna (**k**) with the rotor model and (**l**) without the rotor model when k=2.

A numerical method was applied to analyze the impedance spectrum of the loop antenna integrated with a turbine rotor. Typically, the aperture size of turbine blades is less than one tenth of the wavelength of the loop antenna, operating at several hundreds of megahertz, so the turbine blades can be simply modeled by a stainless-steel disc to accelerate the simulation speed. Because the relative permeability of stainless-steel material is close to one, it would not affect the magnetic field distribution of the loop antenna. Electromagnetic waves are shielded inside the turbine rotor, so only its surface current distribution needs to be solved. The diagrams in Figure 3a show the projection view of the simulation model, and Figure 3b shows a three-dimensional view. The diameter of the turbine blades is defined as twice the diameter of the turbine rotor; other geometric parameter definitions and values in this simulation model are described in Table 2. 

In the simulation model, the rotor height (h) and rotor radius (c) are normalized by the radius of the loop antenna (a) because the influence of the metal environment on the propagation of the electromagnetic wave is determined by the ratio of its physical size to the wavelength, while the antenna radius (a) is proportional to the wavelength according to Equation (4). 

The eddy current on the surface of the turbine rotor model and the magnetic fields distributed at k=2 are displayed in Figure 3b,k to verify the simulation model. The magnetic field distribution of the loop antenna in free space with the same scale of color key is displayed in Figure 3l for contrast. The current and magnetic fields mainly exist near the loop antenna on the rotor and blades, and they are rarely distributed on the rotor away from the antenna and in the volume of the rotor model. It indicates that the turbine rotor is correctly modeled for analyzing the behavior of the loop antenna. It can also be inferred from these figures that the turbine blades will affect the impedance and radiation pattern of the loop antenna, while the rotor height is not a critical parameter because it does not affect the field distribution. This inference is verified by simulations and discussions in the following paragraphs. 

The curves in Figure 3c–e illustrate the relationship between antenna radiation resistance and normalized rotor height (h/a) at different values of normalized rotor radius (c/a). In order to compare these with the loop antenna in free space, the results of simulated radiation resistance are normalized by the values in Table 1. All four figures with k=1,2,3,4 present in a horizontal strip distribution, meaning that the antenna radiation resistance is not sensitive to the height of the turbine rotor. The antenna radiation resistance presents a gradually decreasing trend with the growth of c/a, showing that the rotor radius is the main factor determining the antenna radiation resistance.

Because the rotor height (h) does not have a significant impact on the antenna resistance, it was then fixed to 50 cm for further simulation. The curves in Figure 3g,h illustrate the relationship between the antenna radiation resistance, the self-resonant frequency, and the normalized diameter (c/a), respectively. The antenna frequency is normalized by the simulated values in Table 1. The curves in Figure 3h prove that the antenna frequency changes little for all values of k during the normalized diameter, varying in a wide range. This small amount of change is negotiable during the loop antenna design procedure. Furthermore, comparing the positive frequency error between the estimation and simulation results in Table 1, the decreasing trend of antenna frequency in Figure 3h can offset the estimation error, making the empirical formula in Equation (4) more accurate, especially at larger normalized diameter (c/a) values. 

Figure 3g gives the best design interval for optimizing the loop antenna resistance to 50 Ω without any impedance matching components. All points located in the black limited line window have a VSWR of less than 2 for the 50 Ω reader hardware system. Thus, the optimization goal is realized by fully utilizing the relationship between the radius of the turbine rotor and the antenna radiation resistance.

The return loss ($S_{11}$ parameter) and input impedance ($Z_{11}$ parameter) spectrum of a loop antenna with k=2, λ=75 cm and c/a=0.7 were simulated and are shown in Figure 3i, exhibiting the minimum of the $S_{11}$ parameter to be lower than −10 dB, i.e., VSWR <2. Its self-resonant point appears at 402.5 MHz, which has only a 0.6% error compared to the 400 MHz design value. This again proves the accuracy of Equation (4) for estimating the self-resonant frequency of the loop antenna integrated with a turbine rotor. The estimation error of Equation (4) for other values of normalized diameter (c/a) and k are displayed in Figure 3j, where the minimum error is less than 0.3%, while the maximum error at c/a≥0.6 and k>1 are less than 2%. Moreover, the $S_{11}$ parameter curve in this figure shows a large bandwidth compared to the temperature sensitivity coefficient, which is discussed in Section 3, indicating that the bandwidth of the proposed loop antenna is not a critical design parameter.

The design procedure of the loop antenna integrated with a turbine rotor can be concluded as the inverse process of the above analysis. The radius of the turbine rotor is the only parameter known at first. A normalized diameter (c/a) value in the range of the VSWR window is then chosen by referring to the curves in Figure 3g; thus, the radius of the loop antenna can be denormalized. By selecting different values of k, the operating frequency of the loop antenna, i.e., the system operating frequency, is finally determined according to the estimation formula in Equation (4). Ultimately, parameter k is a coarse adjustment knob for tuning the antenna frequency to an approximate band, while the normalized diameter (c/a) is a fine adjustment knob for tuning the antenna frequency to accurately match the resonant frequency of the SAW sensor tag. The antenna frequency can be arbitrarily modified as long as c/a is in the range of the VSWR window.

The temperature drift and high-temperature performance of the loop antenna impedance should be taken into consideration for wide temperature range operation. Due to the air media of the loop antenna, its temperature drift is mainly caused by the conductivity change of the antenna material. All the simulations above use copper as antenna material, the conductivity of which is approximately 58 MS at room temperature. For higher temperature usage, copper can be replaced by other materials with high temperature tolerance, such as stainless steel, nickel, etc. According to the default material library of HFSS, the conductivity of stainless steel and nickel at room temperature is 1.1 MS and 14.6 MS, respectively. Repeated simulations were conducted to research the conductivity of different materials and the impact of the conductivity reducing at higher temperatures. According to [24,25], the high-temperature conductivity of stainless steel and pure nickel is 1.0 MS (at 600K) and 2.8MS (at 800 K), respectively. Hence, these two values were chosen in the simulation. Model parameters in these simulations are the same as those in Figure 3i, except for the material conductivity. The simulated antenna frequency and radiation resistance are logged in Table 3. The frequency difference (Δf) and resistance difference (ΔR) compared to that at room temperature are also logged.

All these simulated frequencies show a decreasing trend with the reduction of material conductivity, but the change of Δf is very small compared to the antenna bandwidth shown in Figure 3i, which indicates that these materials for high temperature usage have nearly no impact on the antenna frequency, and the temperature drift of the loop antenna is insignificant. Hence, the proposed loop antenna integrated with a turbine rotor can operate stably within a wide temperature range. 

#### 2.1.3. Efficiency and Radiation Patten of the Loop Antenna

In addition to the impedance, the radiation pattern and radiation efficiency of the loop antenna were analyzed to evaluate its performance. The radiation efficiency is determined by the ratio of loss resistance to radiation resistance. The loss resistance is the summation of direct-current (DC) loss and skin-effect loss at high frequency. The former is negotiable, for it is very small compared to the latter. The skin-effect loss resistance of the loop antenna can be calculated by Equation (6):

(6) $$R_{skin}=\frac{a}{b}\sqrt{\frac{\pi f\mu_{0}}{\sigma }}$$

where $\sigma ,\;\mu_{0},\;f$ are the material conductivity, the vacuum permeability, and the operation frequency of the loop antenna. It was calculated that the $R_{skin}$ for the simulation model in Figure 3i is ~ 0.9 Ω, which is small compared to 50 Ω radiation resistance, while its DC loss is hundreds of times smaller. It indicates that the high radiation efficiency of the loop antenna is maintained. 

Attention should be paid to the trade-off between the miniaturization factor of the SAW sensor tag and the loss (i.e., wireless sensing range). The larger k in Equation (4) reduces the value of λ, and thus enables better miniaturization design for the SAW sensor tag, but the loss also increases, including $R_{skin}$ and the propagation loss of the EM wave according to the Friis equation. A larger value of β or multiple parallel loops insulating each other should be used in these cases to decrease the loss in resistance.

**Figure 4:**
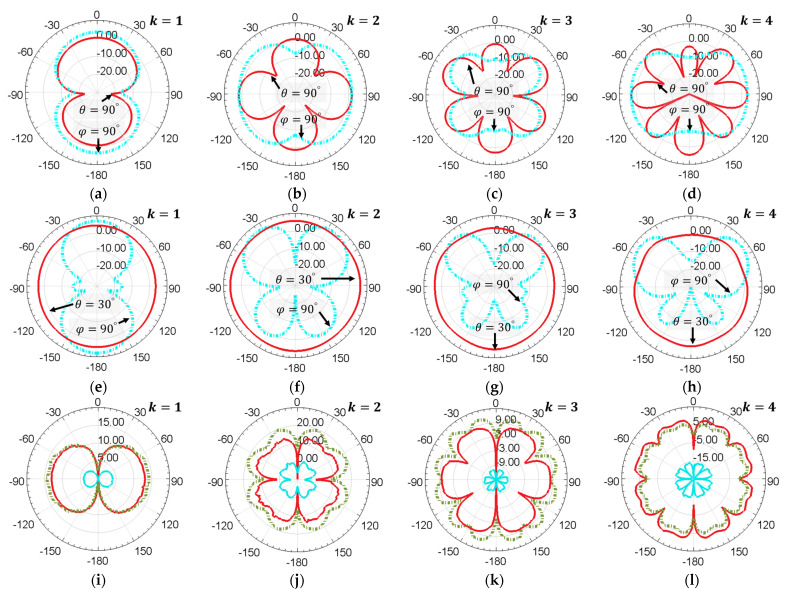
Far-field antenna gain on azimuth angle (red solid line) and elevation angle (blue dot-dash line) of the loop antenna in free space (**a**–**d**) and the loop antenna integrated with a turbine rotor (**e**–**h**) at k=1,2,3,4; (**i**–**l**) Near-field Poynting energy flow at k=1,2,3,4 on three different mounting positions for the SAW sensor tag: on the surface of the turbine rotor close to the loop antenna (green dot-dash line); on the top surface of the turbine blades (red solid line); on the bottom surface of the turbine blades (blue solid line).

Typically, SAW sensor tags can be placed in both the near-field and far-field regions of the loop antenna; hence, the antenna far-field gain and near-field Poynting energy flow are analyzed in Figure 4. The impact of the turbine rotor on the radiation pattern of the loop antenna was demonstrated by comparing that with the loop antenna in free space.

The far-field gain in decibel units on the azimuth angle (red solid line) and the elevation angle (blue dot dash line) of the loop antenna are displayed in Figure 4a–h. The radiation pattern of the loop antenna in the free space at k=1 in Figure 4a is complementary to that of a dipole antenna. The radiation patterns for the loop antenna in free space in Figure 4a–d show that with the increase in k, more sidelobes appear due to the multiple oscillation cycles of current distribution on the loop antenna. The radiation patterns for the loop antenna with turbine rotor in Figure 4e–h show that the antenna beam angle becomes larger with the increase in k due to the sidelobes merging together. Comparing the radiation patterns in Figure 4a–d, the turbine rotor and blades increase the antenna gain and directivity. The value of the maximum far-field gain and its position in spherical coordinate units are listed in Table 4, demonstrating that the gain of the loop antenna integrated with a turbine rotor is several decibels higher than the antenna in free space. 

According to these results, it can be inferred that the turbine blades act like the reflector in a Yagi-Uda antenna. The radius of turbine blades is larger than that of the loop antenna, making the turbine blades an inductive load, the surface current of which lags in terms of voltage; hence, the forward gain is increased. However, different from the traditional Yagi-Uda antenna, the max gain of which appears at θ=90°, the max gain of the loop antenna appears within a range of θ=30° to 60° due to the impact of the turbine rotor. The spacing between the feed element and reflector in the Yagi-Uda antenna is within 0.1 λ to 0.25 λ, which mainly affects the antenna gain, sidelobe pattern, and bandwidth. This spacing corresponds to the distance $h_{1}$ in Figure 3b. If the SAW sensor tag mounted on the rotor is in the far-field region of the loop antenna, this parameter can be further optimized. Even some passive loops can be applied as directors for higher gain, as it will also affect the antenna impedance, which invalidates the look-up curves in Figure 3g. 

For SAW sensor tags mounted in the near-field region of the loop antenna, the Poynting energy flow on the azimuth angle is shown in Figure 4i–l. The data are also displayed in decibel units. Both the top surface of the turbine blades and the surface close to the antenna on the turbine rotor receive large amounts of power, as shown in the red solid line and green dot-dash line, respectively. The blue solid lines shrinking at the center of the polar coordinate illustrate that almost no signal can be received on the bottom surface of the turbine blades due to the shielding of electromagnetic waves.

### 2.2. Optimization for the SAW Senor Tag

Since methodologies for finding the size of the loop antenna and system operating frequency have been proposed and the performance of the loop antenna has been optimized in the above sections, the design of the SAW sensor tag is introduced here.

The SAW sensor tag for metal rotating objects should have the properties of being low weight, low profile, and be highly compact. The geometric size of the SAW sensor tag is mainly determined by its antenna, because the antenna size should be comparable to its electric wavelength at the centimeter level, while the size of the SAW resonator is comparable to its acoustic wavelength at the micrometer level. Therefore, antenna miniaturization is the initial factor to be considered for designing the SAW sensor tag. It is discussed in Section 2.2.1.

Methodologies for designing the SAW temperature sensor in this system are discussed in Section 2.2.2. The geometry parameters of the SAW sensor and the material of its piezoelectric substrate are optimized for good impedance matched with the SAW antenna, high quality factor, and fewer parasitic parameters. Finally, the process flow for fabricating the SAW temperature sensor is reported.

#### 2.2.1. Considerations for the SAW Antenna

Typical antenna types for SAW sensor tags are listed in Table 5, including PIFA, loop coupler, helical, and dipole antenna (spatial or on printed circuit board). Their gain, size, bandwidth, and stability for operating in complex metal environments are compared. More efforts should be made in terms of reducing the antenna size in this application, but the antenna gain and bandwidth are sacrificed. Antenna gain and bandwidth are often two opposing parameters; that is, an antenna with larger gain usually has less bandwidth. But the antenna bandwidth also affects the temperature sensing range of this system; hence, the antenna bandwidth is more important than its gain. Moreover, when this trade-off issue is systematically considered, multiple methods can be adopted for compensating the sacrifice of the SAW antenna gain, such as increasing the quality factor of the SAW resonator and optimizing the loop antenna gain mentioned previously.

**Figure 5:**
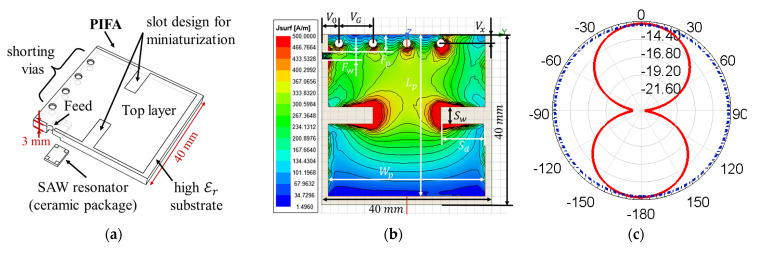

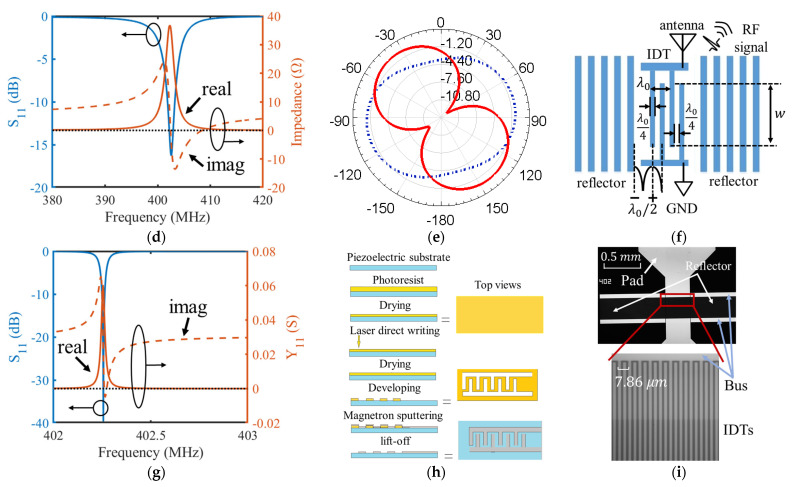
(**a**) Architecture for the SAW sensor tag in this work; (**b**) Geometric parameters of the PIFA together with its simulated surface current distribution; (**c**) Simulated radiation pattern and (**d**) impedance spectrum of the proposed PIFA; (**e**) Tilted radiation pattern with polarization mismatch simulated by the asymmetric PIFA structure in [26] for contrast, where the red solid line represents the azimuth angle, and the blue dashed line represents the elevation angle. (**f**) Geometry size of IDTs and reflectors in the SAW resonator; (**g**) Impedance spectrum of the SAW resonator simulated by p-matrix; (**h**) The processing of laser direct writing; (**i**) Photograph of the SAW resonator under microscope.

In addition to the antenna gain and bandwidth, stability for operating in complex metal environments is also another important consideration in this sensing system. The impedance of the helical antenna and dipole is hard to optimize because it varies a lot in different metal environments, while PIFA works stably in such environments. Furthermore, PIFA is the best miniaturization design among the listed antenna types, although its gain is sacrificed. Although PIFA has less bandwidth than other types of antennas, it is adequate for covering the wide temperature sensing range of our SAW sensor. This is analyzed in detail in Section 3. The design architecture of PIFA used in this work is displayed in Figure 5a, where the top layer graphic was formed by etching on a double-sided copper-clad ceramic substrate and the SAW resonator was embedded in the bottom side of the PIFA substrate. 

Maximizing the current path [26] on the top layer of PIFA is the main approach for antenna miniaturization; this is achieved by the slot design shown in Figure 5a. The adoption of a microwave ceramic substrate with a high dielectric constant ($\varepsilon_{r}=20.0$) further reduces the volume of PIFA, guaranteeing the safer operation of the rotating objects. The total antenna volume in this work after optimization is approximately λ/19×λ/19×λ/250, which is only 7% of that in [26]. The simulated surface current distribution of PIFA is displayed in Figure 5b. The values and explanations of the geometric parameters annotated in Figure 5b are all listed in Table 6. Ceramic material with a higher dielectric constant can further miniaturize the PIFA, but the antenna’s $S_{11}$ parameter will appear as a sharp valley with a small bandwidth, which reduces the temperature sensing range.

The shape and position of the slot design and via array not only influence the miniaturization factor of the PIFA, but they also affect its radiation pattern. The distortion and tilt of the radiation pattern change the polarization direction of EM waves, which can deteriorate the quality of the wireless sensing signal, especially for our system operating in the complex metal environment of the turbine rotor. We optimized the PIFA structure in [26] and improved this performance by creating a symmetrical slot pattern at the center of the radiation surface. Figure 5c illustrates the simulated radiation pattern after optimization with the maximum radiation value occurring at the 0° position. The red solid line represents the azimuth angle, while the blue dot dash line represents the elevation angle. As a comparison, the radiation pattern before optimization has a tilt of ~ 45° to the left in Figure 5e, which leads to a worse polarization match.

The impedance spectrum of the proposed PIFA is simulated in Figure 5d; the $S_{11}$ parameter can be optimized by tuning its feed position. Compared to the impedance spectrum of the PIFA and the loop antenna, the bandwidth of the PIFA is several megahertz smaller than that of the loop antenna. Hence, the temperature drift of the PIFA is more critical. Different from the loop antenna, the temperature drift of the PIFA is mainly caused by the temperature drift of the substrate dielectric constant. The issue was solved by adopting a microwave ceramic substrate, ‘D6D’, with a ~−1 ppm temperature coefficient from cetc-13. The S-parameters of the proposed PIFA are tested in Section 3.

The proposed PIFA structure was also optimized for engineering. The frequency error in the manufacturing process can be easily tuned. Firstly, reducing the number of vias in the array by drilling to destroy the metallization in those vias can coarsely reduce the antenna frequency. Secondly, the slot design in Figure 5a also has merit for tuning the antenna frequency. The current path of the PIFA can be slightly reduced by filling part of the slot using conductive materials, such as silver lacquer, which could increase its frequency. Opposingly, the frequency can be reduced by making the slot longer. Another merit of the PIFA structure is the flat ground plane at its bottom layer, which facilitates the embedding of the SAW sensor and the attachment of the SAW sensor tag onto metal rotating objects.

#### 2.2.2. Design of the SAW Temperature Sensor

The SAW sensor used in this system is a single-port SAW resonator, the geometry size of which is shown in Figure 5f. Both the finger width and the finger gap of its interdigital transducer (IDT) are $\lambda_{0}/4$, where $\lambda_{0}$ is the acoustic resonant wavelength of the SAW equaling the SAW velocity divided by the system operating frequency. Open-circuit reflectors are used in this design according to their standing-wave pattern, as shown in Figure 5f; the gap from the IDT finger to the reflector is $\lambda_{0}/2$. A larger amount of IDT pairs and reflectors has the advantage of having high quality factors, which enhance the wireless sensing distance; however, after reaching a certain level, the effect is no longer significant as it leads to an excessive chip area and a static capacitance [27,28]. It will cause a poor impedance match with the SAW antenna and frequency offset. A total of 150 pairs of IDT and 250 pairs of reflectors were chosen in this work, referring to [28]. After determining the above parameters, the aperture of the SAW resonator (w) is then calculated by the input resistance of the SAW resonator expressed in Equation (7):

(7) $$R_{in}=\frac{1}{2wf_{0}C_{0}}\frac{4k_{t}^{2}}{{\left(4k_{t}^{2}N\right)}^{2}+\pi^{2}}$$

where $R_{in}$ is 50 Ω for typical RF systems, $f_{0}$ is the system operating frequency already determined by the turbine rotor radius, N is the number of IDT pairs, $k_{t}^{2}$ and $C_{0}$ are the material constants of the electromechanical coupling coefficient and unit capacitors, respectively. The design was then verified by simulating the impedance spectrum using the acoustic propagation matrix; the results are presented in Figure 5g.

The piezoelectric substrate material of the SAW sensor is YX-cut quartz for its high quality factor (inversely proportional to $k_{t}^{2}$) and high temperature tolerance (a phase transition temperature of more than 500 °C) compared to lithium niobate ($LiNbO_{3}$) [29]. Its high temperature coefficient also makes this material a good candidate for temperature sensors. For passive wireless measurements of the other physical quantities such as strain, torque, etc.; ST-cut quartz should be chosen due to its zero temperature drift property. 

The laser direct writing (LDW) [30,31] technique was adopted for the fabrication of the SAW resonator for its advantages in terms of being mask-less, its high efficiency, its high resolution, and it being cost-effective. The basic procedures for this technique are described in Figure 5h. Metallization with 150 nm IDT thickness was formed by sputtering using aluminum material for its lightweight and high quality factor. A photograph under a microscope of the SAW resonator fabricated using the LDW technique is shown in Figure 5i.

## 3. Experimental Results and Discussion

**Figure 6:**
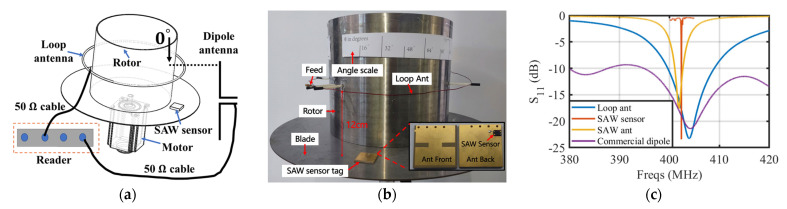

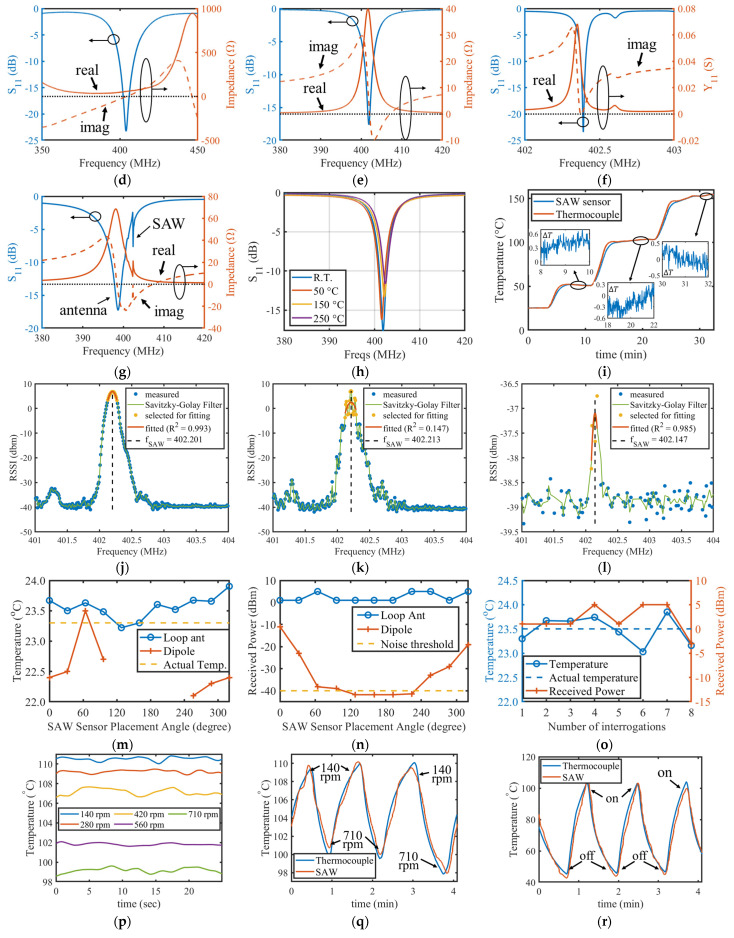
Experimental equipment setup and results: (**a**) Diagram and (**b**) experimental setup of the metallic turbine model; (**c**) S-parameters of used antennas and the SAW sensor; Measured $S_{11}$ and $Z_{11}$ of (**d**) the loop antenna integrated with a rotor model, (**e**) PIFA, (**f**) SAW resonator, (**g**) SAW resonator integrated with PIFA; (**h**) Measured $S_{11}$ of the PIFA at various temperatures; (**i**) Sensing accuracy of the SAW sensor tag over a wide temperature range compared to the thermocouple; RSSI data output of one frequency sweep and its fitting results at (**j**) static test with the proposed loop antenna, (**k**) rotation test with loop antenna, and (**l**) static test with the commercial dipole antenna; (**m**) Temperature and (**n**) power measured at different placement angles during the static test; (**o**) Eight repeat experiments of temperature and power measured during the dynamic test of the turbine model; The temperature measured by the SAW sensor tag under constant power heating (**p**) at different rotational speeds and (**q**) step changes in rotational speed from 140 rpm to 710 rpm; (**r**) The temperature measured by the SAW sensor tag during periodic switching of the heater at a rotational speed of 560 rpm.

The experimental setup in order to verify the design of the loop antenna and the EM shielding problem of the system in a rotating metal environment is shown in Figure 6a,b, where Figure 6a is the equipment for dynamic temperature sensing with the rotation of the rotor and Figure 6b is the equipment for static temperature sensing. The stain-less steel barrel and disc for modeling the turbine rotor and blades are the same size as the simulation model in Figure 3i. An angle scale was attached to the rotor for indicating the relative position of the antenna and the SAW sensor. A comparative experiment was also conducted by using a commercial 400 MHz dipole antenna placed at 0 degrees of the angle scale. 

The $S_{11}$ parameters of the loop antenna, SAW sensor, SAW antenna, and commercial dipole antenna were initially measured using a vector network analyzer (E5080B manufactured by Keysight in California, USA) to verify the correctness of the theoretical calculations and simulation results discussed in Section 2. The valleys of the $S_{11}$ curves in Figure 6c all match near 402 MHz and agree well with the simulations. The impedance spectrum of the loop antenna integrated with the rotor model, PIFA, SAW resonator, and SAW sensor tag (SAW resonator integrated with the PIFA) are displayed in Figure 6d–g, respectively, and they are compared with the simulation results in Figure 3i and Figure 5d,g. 

It was found that the measured impedance spectrum of the SAW resonator had some parasitic peaks that were not presented in the simulation, and its quality factor was lower than that in the simulation when compared to the bandwidth of the S-parameter curves. Figure 5g indicates that the PIFA frequency was lowered by the parallel connection of the parasitic capacitance in the SAW resonator, but this did not affect the operation of the SAW sensor tag as the proposed PIFA structure was optimized for the flexibility of tuning. Ultimately, the difference between the simulated and measured results is tolerable, as they have little impact on the system design methodologies proposed in this paper. 

The loop antenna has a measured bandwidth of ~9 MHz, which is more than 10 times larger than the maximum Δf caused by the temperature drift in Table 3, so it can be treated as a zero temperature drift within a wide temperature range. The SAW antenna has a smaller bandwidth of ~2 MHz due to the trade-off between its miniaturization design. Benefiting from its substrate material, the PIFA is nearly a zero temperature drift according to the measured $S_{11}$ curves in Figure 6h. The resonant frequency of the SAW sensor on the YX-cut quartz has a temperature coefficient of approximately +10 kHz/°C (+25 ppm/°C) and the maximum temperature sensing range is estimated by the minimum antenna bandwidth in the system divided by the sensitivity of the SAW sensor, which is approximately 200 °C in this system. 

The SAW sensor tag was then calibrated using a thermocouple, and a heating experiment up to 155 °C was conducted to verify its accuracy. The temperature variation during the heating process is presented in Figure 6i; the temperature difference between the two curves was calculated in its subplots when the temperature became stable. These experimental results demonstrate that the sensing error is less than 1 °C in the measured range.

The frequency-sweeping curves of the RSSI output by the reader are displayed in Figure 6j–l, where the blue dots are raw RSSI data at every frequency point. The noise floor of these raw data is approximately −40 dBm. The green solid lines are these same raw data smoothed by a Savitzky-Golay filter with a frame length of 5 and weights of 7 for higher accuracy. The yellow points are data selected for fitting using a quadratic polynomial, and the black dashed lines are fitting results for the resonant frequency of the SAW sensor ($f_{SAW}$). This fitting result corresponds to the vertex position of the fitted parabola displayed by the red solid lines in these figures. The fitted frequency $f_{SAW}$ is then converted to temperature data by linear transformation and averaged for noise reduction.

Static wireless temperature measurement at room temperature and different placement angles of the SAW sensor tag corresponding to the angle scale on the rotor were conducted to verify the system. The measured temperature data and peak power of the RSSI with different rotating angles using the proposed loop antenna in this system and the commercial dipole antenna are shown in Figure 6m,n, respectively. The results show that the loop antenna can achieve continuous reading of the temperature at arbitrary angles with an error of less than 1 °C, owing to its stable sensing signal. The low ripple of the received power helps to reduce the temperature measurement error. Compared with the conventional dipole antenna, its signal was shielded at the back of the rotor, resulting in discontinuous sensing. The received power of the dipole antenna shows large fluctuations and is even drowned by the noise level when the sensor is moved to the back of the turbine model. Figure 6j,l are the RSSI data of one frequency sweep at a 60° placement angle for the loop antenna and commercial dipole antenna, respectively. The former data present as a smooth parabola with very good signal strength, while the later data are nearly drawn into the noise floor, which causes inaccuracy at fitting the $f_{SAW}$.

A dynamic test at room temperature was then carried out by clamping the SAW sensor on a tripod while rotating the barrel with a speed of 60 rpm. Figure 6o shows the logged temperature and peak power of the RSSI at eight repeated integrations, which also has an error of less than 1 °C and a low ripple RSSI curve. The RSSI data of one frequency sweep test corresponds to the curves in Figure 6k. Fluctuations appear at the vertex of the parabola due to the vibration of rotation and the sidelobes of the antenna radiation pattern, which causes the fitting coefficient of determination ($R^{2}$) to drop to a low value. Nevertheless, the accuracy of the measured temperature is not affected, owing to its large signal strength. Meanwhile, filters applied to the RSSI data further reduce the fluctuations during rotation.

To further verify the performance of the proposed passive wireless sensing system, we conducted experiments at higher rotational speeds and temperatures. First, we applied constant power heating to the SAW sensor tag and adjusted the rotational speed of the rotor. After the temperature stabilized, we performed wireless temperature readings, and the results are shown in Figure 6p. It can be observed that as the rotational speed increases, heat dissipation accelerates, causing the temperature on the sensor to gradually decrease. These curves also demonstrate that the sensor exhibits good temperature measurement fluctuations, generally ~1 °C, indicating that the system proposed in this paper can operate stably under these rotational speeds. 

On the other hand, we also used constant power heating for the SAW sensor tag and periodically adjusted the rotational speed between 140 rpm and 710 rpm during operation, as shown in Figure 6q. It can be seen that the temperature rises and falls in line with the periodic changes in heat dissipation rate at different rotational speed. In the experiment, wired thermocouple measurements were used as a contrast, and it is evident that the temperature variation trends of the SAW sensor tag closely match those of the thermocouple, with good accurate measurements. In Figure 6r, we operated the experimental setup at a constant rotational speed of 560 rpm and periodically switched the heating power on and off. The temperature measurements from the proposed sensing system remained consistent with the thermocouple readings throughout.

## 4. Conclusions

In summary, we proposed a passive wireless SAW sensing system with a wide operating temperature range and solved the EM shielding problem for rotating metal parts. The loop antenna with omnidirectional radiation pattern helps this system achieve real-time and continuous monitoring of the working status of rotating metal parts. 

Systematical methodologies for the optimization of the loop antenna and the SAW sensor tag were concluded according to the simulation results and theoretical analysis. The performance of the system components was collaboratively analyzed and designed, such as the antenna bandwidth, gain, temperature drift, and the system temperature sensing range. By combining the simple empirical formula and look-up curves with the VSWR window proposed in this paper, a loop antenna integrated with a turbine rotor can be quickly designed in different wave bands with continuously tunable frequencies. According to the simulation results, the empirical formula has a minimum estimation error of less than 0.3%.

The correctness of the proposed turbine rotor model was verified by analyzing the distribution of eddy currents on the model surface and magnetic fields around the antenna. The simplicity of the rotor model facilitates the establishment of an experiment environment, and it can also improve the simulation efficiency.

The main performance of the loop antenna integrated with a rotor, including the antenna gain and antenna impedance, were simulated and optimized. The antenna radiation resistance was optimized to 50 Ω without any additional impedance matching components by utilizing the impact of the metal rotor on antenna impedance, which enabled the proposed loop antenna to operate stably in high-temperature environments. The temperature drift of the loop antenna frequency and radiation resistance was simulated by modifying its material conductivity and was proven to be negligible. The turbine blades were utilized as the reflector, hence improving the far-field gain of the loop antenna, which increased the wireless sensing distance for the SAW sensor tags mounted in the far-field region. The near-field Poynting energy flow was also analyzed to determine the best mounting position for the SAW sensor tag in the near-field region. 

Methodologies for designing the SAW sensor tag were discussed in this paper. The proposed PIFA structure was optimized for miniaturization and had the properties of a matched polarization direction and easy tuning at the same time. Benefiting from its substrate material with a very low temperature coefficient, the PIFA frequency was stable within its operating temperature range according to the measurement results. Key parameters and procedures of fabricating the SAW resonator for optimized performance were introduced, including the choice of piezoelectric substrate material, the determination of IDT finger numbers, and the metal material. The high-accuracy and low-cost LDW technique provided by our micro/nano fabrication center simplified and accelerated the iteration process of designing the SAW resonator for temperature sensing.

The system was experimentally verified with a metallic turbine model. The test results demonstrated that the proposed loop antenna can greatly improve the stability of the sensing signal when the SAW sensor tag is rotating. The variance of the measured temperature data in the rotating experiment was within 1 °C, providing promising applications in engines and other rotating scenarios. In contrast, the traditional dipole antenna could not receive the sensing signal at the back of the rotor. 

The same accuracy is maintained when the SAW sensor tag is heated from room temperature to 155 °C. The accuracy of the passive wireless SAW sensing system is often reduced due to the Doppler frequency shift of rotating objects, but in this work, the Doppler effect does not exist because the relative position of the SAW sensor tag and loop antenna is fixed. Therefore, the proposed sensing system could have good potential for applications in metal rotating parts with higher speeds. This was further confirmed in subsequent experiments, where the proposed rotational setup was able to achieve accurate passive wireless temperature sensing at a rotational speed of 710 rpm.

Above all, this work demonstrated that the proposed loop antenna and SAW sensor tag structure are simple yet effective systematical solutions for real-time status monitoring of rotating metal parts.

## Figures and Tables

**Table 1:** Simulated frequency and resonant resistance of the loop antennas in free space.

k	Designed Frequency (MHz)	Simulated Frequency (MHz)	Simulated Radiation Resistance (Ω)	Frequency Error (%)
1	400	426.35	156.60	+6.59
2		420.35	205.46	+5.09
3		417.35	228.14	+4.34
4		416.15	251.35	+4.04

**Table 2:** Parameter definition of the simulation model.

Parameter Symbols	Definitions	Values for Simulation
c	Radius of the turbine rotor	Normalized by antenna radius
h	Height of the turbine rotor	
$h_{1}$	Distance between the loop antenna and turbine blades	Fixed at 15 cm
β	$\beta =2\ln\left(2\pi a/b\right)$	Fixed at 14
λ	Antenna wavelength	Fixed at 75 cm

**Table 3:** Change of frequency and radiation resistance with reduced material conductivity.

Material Conductivity (MS/m)	k	Simulated Frequency (MHz)	Δ*f* (MHz)	Simulated Radiation Resistance (Ω)	Δ*R* (Ω)
1.0 (For stainless steel at high temperature)	1	405.28	−0.70	15.99	+3.20
	2	402.46	−0.38	45.21	+2.60
	3	401.12	−0.24	83.47	+2.79
	4	400.90	−0.18	108.42	+2.72
2.8 (For nickel at high temperature)	1	405.60	−0.38	14.53	+1.73
	2	402.64	−0.21	44.02	+1.41
	3	401.23	−0.13	82.15	+1.47
	4	400.98	−0.10	107.20	+1.50

**Table 4:** Maximum far-field gain and its position for the models in Figure 4a–h.

Simulation Model	k	Max Gain (dB)	Max Gain Position
Loop antenna in free space in Figure 4a–d	1	3.46	φ=0°, θ=180°
	2	3.00	φ=180°, θ=42°
	3	4.59	φ=180°, θ=50°
	4	5.75	φ=180°, θ=58°
Loop antenna integrated with turbine rotor in Figure 4e–h	1	7.76	φ=180°, θ=180°
	2	6.55	φ=180°, θ=36°
	3	6.97	φ=180°, θ=44°
	4	7.36	φ=180°, θ=48°

**Table 5:** Performance comparison for typical antenna types of SAW sensor tag.

Antenna types	Gain	Size	Bandwidth	Metal Environment
PIFA	Low	Small	Medium	Good
Loop coupler	Very low	Medium	Medium	Good
Helical	Medium	Medium	Wide	Poor
Dipole	High	Large	Wide	Poor

**Table 6:** Geometric parameters of the PIFA annotated in Figure 5b for simulation.

Parameters	Value	Explanation
$V_{0}$	4 mm	Distance from the first via to the edge of the substrate
$V_{G}$	8 mm	Gap between the vias
$V_{x}$	1 mm	Distance from via array to the edge of the substrate
$F_{w}$	2 mm	Feed width
$F_{p}$	4 mm	Feed position
$S_{w}$	4 mm	Slot width
$S_{d}$	10.5 mm	Slot depth
$L_{p}$	38 mm	Total length of the top layer graphic
$W_{p}$	37 mm	Total width of the top layer graphic

## Data Availability

The data that support the findings of this study are available from the corresponding author upon reasonable request.
